# Contemporary Theory and Research

**Published:** 1893-05-20

**Authors:** 


### CONTEMPORARY THEORY AND RESEARCH.

### THE EPILEPTIC COLONY.

The *Journal of Mental Science* has a brief article advocating the proposed home for those necessitous epileptics who are able and willing to work. "Those subject to epileptic fits being by the rules of many homes excluded from them, the want of employment and the consequent worries of poverty aggravate their malady, and the utter helplessness of their condition leads to despondency and hastens their descent towards dementia. There are nearly 78,000 epileptics in Great Britain, and 39,000 of these are still in the full possession of their reason. Now it is a fact that the condition of life most calculated to ward off epileptic attacks is that of healthy occupation. It is proposed to secure an estate of 100 acres within fifty miles of London and to erect upon it appropriate buildings. The occupation provided will take the form of farm and garden work, which is at once easy and healthy. Gradually other and more varied work will be provided. Wealthy cases will be provided for and freely admitted, and to a great extent it is expected that the colony will become self-supporting."

### MENTAL CHRONOMETRY.

Dr. Baldwin contributes to the *Medical Record* a note on the Psychology of Reaction time. "The recognition of the great forms of aphasia, *i.e.*, sensory and motor, and the corresponding recognition of the existence of visual, auditory, and motor speech types, gives a strong presumption that the distinction between sensory and motor in the voluntary movements of speech and writing applies as well to voluntary movements of all kinds; that is to all movements which have been learned by attention and effort. This means that a man is an 'auditive' or a 'visual,' or a 'motor' in his voluntary movements generally. His attention is trained by habit, education, &c., more upon one class of images than upon others, his mind fills up more easily with images of this class, and his mental processes and voluntary reaction proceed by preference along these channels of easiest functions. If this be true, it is evident a man's reaction time will show the influence of his memory type. The motor reaction we should expect to be most abbreviated in the man of the motor type; and less abbreviated or not so at all in the 'visual' or 'auditory' man. The position is in direct accord with Pinch's interesting argument for the central seat of the motor disturbances which result in certain cases of anæsthesia from the closure of the eyes. It is really the attention which is disturbed in these cases through the loss of its usual support from the sense of sight; it is not a loss of muscle sense only. In addition, the indications of memory type afforded by reaction-times also support the analysis of speech from aphasic cases. A man with a relatively short 'sensory' reaction will be of the 'sensory' type, and will be peculiarly liable to 'sensory' forms of aphasia—loss of speech through word blindness, word deafness, &c., and to paraphesia and paragraphia. On the other hand, one whose 'motor' reaction time is very short will be liable to loss of speech from interference with his muscular memories. I am not sure that such a correspondence could be made out in actual cases of aphasia, but it is an interesting deduction, and possibly medical men may find opportunity of testing it with the aid of a portable instrument, such as the 'Chronomètre d'Arsonval.'"

